# Integrated analyses reveal two molecularly and clinically distinct subtypes of H3 K27M-mutant diffuse midline gliomas with prognostic significance

**DOI:** 10.1007/s00401-024-02800-3

**Published:** 2024-09-10

**Authors:** Lotte Stegat, Alicia Eckhardt, Antonia Gocke, Sina Neyazi, Lara Pohl, Simone Schmid, Matthias Dottermusch, Stephan Frank, Hans Pinnschmidt, Jochen Herms, Markus Glatzel, Matija Snuderl, Leonille Schweizer, Christian Thomas, Julia Neumann, Mario M. Dorostkar, Ulrich Schüller, Annika K. Wefers

**Affiliations:** 1 Institute of Neuropathology, University Medical Center Hamburg-Eppendorf, Hamburg, Germany; 2 Research Institute Children’s Cancer Center Hamburg, Martinistrasse 52, N63 (HPI), 20251 Hamburg, Germany; 3 Center for Molecular Neurobiology Hamburg, University Medical Center Hamburg-Eppendorf, Hamburg, Germany; 4 Section of Mass Spectrometric Proteomics, University Medical Center Hamburg-Eppendorf, Hamburg, Germany; 5 Department of Pediatric Hematology and Oncology, University Medical Center Hamburg-Eppendorf, Hamburg, Germany; 6 Department of Neuropathology, Charité - Universitätsmedizin Berlin, Corporate Member of Freie Universität Berlin, Humboldt-Universität zu Berlin, and Berlin Institute of Health, Berlin, Germany; 7 German Cancer Consortium (DKTK), Partner Site Berlin, and German Cancer Research Center (DKFZ), Heidelberg, Germany; 8 Department of Neuropathology, Institute of Pathology, Basel University Hospital, Basel, Switzerland; 9 Institute of Medical Biometry and Epidemiology, Center for Experimental Medicine, University Medical Center Hamburg-Eppendorf, Hamburg, Germany; 10 Center for Neuropathology and Prion Research, Ludwig-Maximilians-University, Munich, Germany; 11 Department of Pathology, NYU Langone Medical Center, New York, USA; 12 Edinger Institute (Institute of Neurology), University Hospital Frankfurt, Goethe University, Frankfurt am Main, Germany; 13 German Cancer Consortium (DKTK), Partner Site Frankfurt-Mainz, German Cancer Research Center (DKFZ), Heidelberg, Germany; 14 Frankfurt Cancer Institute (FCI), Frankfurt am Main, Germany; 15 Institute of Neuropathology, University Hospital Münster, Münster, Germany; 16 Karl Landsteiner Privatuniversität für Gesundheitswissenschaften, St. Pölten, Austria; 17 Mildred Scheel Cancer Career Center HaTriCS4, University Medical Center Hamburg-Eppendorf, Hamburg, Germany; 18 Present Address: Department of Neuropathology, Regensburg University Hospital, Regensburg, Germany

**Keywords:** Diffuse midline glioma, Survival, DNA methylation analyses, Mutations

## Abstract

H3 K27M-altered diffuse midline gliomas (DMGs) are highly malignant tumours that arise in the midline structures of the CNS. Most DMGs carry an H3 K27M-mutation in one of the genes encoding for histone H3. Recent studies suggested that epigenetic subgroups of DMGs can be distinguished based on alterations in the MAPK-signalling pathway, tumour localisation, mutant H3-gene, or overall survival (OS). However, as these parameters were studied individually, it is unclear how they collectively influence survival. Hence, we analysed dependencies between different parameters, to define novel epigenetic, clinically meaningful subgroups of DMGs. We collected a multifaceted cohort of 149 H3 K27M-mutant DMGs, also incorporating data of published cases. DMGs were included in the study if they could be clearly allocated to the spinal cord (*n* = 31; one patient with an additional sellar tumour), medulla (*n* = 20), pons (*n* = 64) or thalamus (*n* = 33), irrespective of further known characteristics. We then performed global genome-wide DNA methylation profiling and, for a subset, DNA sequencing and survival analyses. Unsupervised hierarchical clustering of DNA methylation data indicated two clusters of DMGs, i.e. subtypes DMG-A and DMG-B. These subtypes differed in mutational spectrum, tumour localisation, age at diagnosis and overall survival. DMG-A was enriched for DMGs with MAPK-mutations*,* medullary localisation and adult age. 13% of DMG-A had a methylated *MGMT* promoter. Contrarily, DMG-B was enriched for cases with *TP53*-mutations, *PDGFRA*-amplifications, pontine localisation and paediatric patients. In univariate analyses, the features enriched in DMG-B were associated with a poorer survival. However, all significant parameters tested were dependent on the cluster attribution, which had the largest effect on survival: DMG-A had a significantly better survival compared to DMG-B (*p* < 0.001). Hence, the subtype attribution based on two methylation clusters can be used to predict survival as it integrates different molecular and clinical parameters.

**Supplementary Information:**

The online version contains supplementary material available at 10.1007/s00401-024-02800-3.

## Introduction

Diffuse midline gliomas (DMGs) are highly malignant tumours that arise in the midline structures of the central nervous system [37]. They are most frequently located in the pons or thalamus but can also be found in other midline structures like the spinal cord [5]. DMGs are thought to originate from oligodendrocyte precursor cells [15, 21]. Depending on the localisation, median age of patients is about 11‒20 years, but DMGs also occur in adults [28].

*MGMT*-promoter methylation predicts a better response to the drug temozolomide (TMZ) compared to tumours with an unmethylated *MGMT*-promoter [20]. Even though *MGMT* promoter methylation was detected in only 0‒4.5% of DMGs [2, 23, 26], patients with DMGs are routinely treated with TMZ [39]. Whilst novel therapeutic concepts such as a CAR-T cell therapy [27] and an H3 K27M-targeted vaccination [19] may significantly improve survival in the future, the prognosis is currently still poor with a two-year survival rate of less than 10% [26]. Hence, these highly aggressive tumours are graded as CNS WHO grade 4.

Genetically, DMGs typically have a mutation in one of the highly homologous genes encoding for histone H3 [35, 40]*.* The mutations lead to the exchange of the amino acid lysine to methionine in position 28 of the amino acid tail (p.Lys28Met, “H3 K28M*”*; former nomenclature, used in the WHO classification, H3 K27M [24]). The majority of DMGs has a K27M-mutation in the *H3-3A* gene, while H3-C2 K27M is much less frequent and most abundant in pontine DMGs [10, 26]. Mutant H3 K27M inhibits the Polycomb repressive complex 2 (PRC2) through interaction with the histone-lysine N-methyltransferase “enhancer of zeste homolog 2” (EZH2). Consequently, this leads to a global reduction of histone H3 trimethylation, resulting in an epigenetic modification that contributes to the tumourigenesis of DMGs [3, 25]. Rarely, DMGs lack an H3 K27M mutation but show an alteration of *EGFR* [36] or an *EZHIP* overexpression [8]. Additional mutations frequently occur in the Tumour Protein 53 (*TP53*)*,* Neurofibromin (*NF1*) and alpha-thalassemia/mental retardation syndrome X-linked (*ATRX*) genes [34, 35].

To define subgroups of CNS tumours that may be clinically or therapeutically relevant, global DNA methylation profiling is often used [7]. Recent studies on DMGs have pursued different hypotheses how subgroups of DMGs may be defined, each focussing on a distinct feature. Castel and colleagues showed that pontine DMGs epigenetically subdivide according to the mutant H3 genes [10]. Chen and colleagues studied the localisation and demonstrated that to some extent, pontine and medullary DMGs may be separated with DNA methylation analyses [12]. Recent studies indicated that DMGs that have mutations in genes associated with the mitogen-activated protein kinases (MAPK) pathway, which regulates tumour cell proliferation and survival [29], may constitute a new subtype of DMG with a longer overall survival (OS) [1, 32, 33]. However, it is currently still unclear how the different parameters that were studied individually, i.e. tumour localisation, patient age and different mutations, interact and collectively influence survival. To integrate different molecular and clinical features in an unbiased manner, we set up a large multifaceted DMG cohort of 149 cases, including data of published cases. The only inclusion criteria were that the tumours had a distinct localisation (spinal, medullary, pontine or thalamic), and that basic clinical data as well as tissue for DNA methylation analyses or DNA methylation data were available. We then comprehensively characterised DMGs, integrating epigenetic data, the mutational spectrum as well as localisation and clinical data, to define novel epigenetic, clinically meaningful subtypes of DMGs.

## Materials and methods

### Cohort

Twenty-eight samples of DMGs were collected from the Institute of Neuropathology at the University Medical Center Hamburg-Eppendorf (UKE) and neuropathological institutions in Basel, Berlin, Münster, and Munich, and further processed at the UKE. Additional data (DNA methylation, sequencing and clinical data) were collected from published data of 121 DMGs [7, 10, 12, 17, 36, 37].

### DNA-methylation data

DNA was isolated using the RSC FFPE Plus DNA Kit (Promega) according to manufacturer’s instructions. 100–500 ng DNA was used for bisulfite conversion by the EZ DNA Methylation Kit (Zymo Research). The DNA Clean and Concentrator-5 (Zymo Research) and the Infinium HD FFPE DNA Restore Kit (Illumina) were employed to clean and restore the converted DNA. DNA methylation data were obtained using the Illumina HumanMethylation450 BeadChip or Infinium MethylationEPIC BeadChip (Illumina, San Diego, California, USA). The DKFZ/Heidelberg brain tumour classifier v11b4 (https://www.molecularneuropathology.org) was used for the verification of the diagnosis of an H3 K27M-mutant DMG. Only DMGs with a classifier score ≥ 0.7 were included in the study. All cases were reassessed later using the updated classifier version v12.8. DMGs with *EGFR* alterations or *EZHIP* overexpression were excluded.

Data were analysed with R [38] in RStudio as described previously [6]. Potential batch effects caused by differences between FFPE samples and frozen tissue were corrected using the *limma* package. Unsupervised hierarchical clustering was done with the *ComplexHeatmap* package (10,000 most variable CpGs, Euclidean distance, Ward.D2 linkage). In addition, we performed *k*-means clustering to verify the results from unsupervised hierarchical clustering with a different method. The optimal number of clusters was determined using the *Elbow* and the *Silhouette methods* using the R-packages *factoextra, tidyverse* and *cluster*. Uniform Manifold Approximation and Projection (UMAP) for dimension reduction was done using the *umap* package (10,000 most variable CpGs). Cases from Capper et al. [7], Ghasemi et al. [18], Pohl et al. [30], Raffeld et al. [31], Sievers et al. [36] were used as reference cases.

### DNA sequencing

DNA sequencing was either done by Sanger sequencing with standard protocols or using different panels (QIAseq CDHS-21330Z-424, Qiagen, Hilden, Germany; LMU Brain Tumour Panel [4]; TruSight Oncology 500, Illumina, San Diego, California, USA) according to the manufacturers’ instructions. Sequencing was done on an Illumina MiniSeq or NextSeq 550 sequencing system. Data were analysed with the Qiagen CLC Genomics workbench or with self-customised workflows. A set of 14 genes was further analysed (*H3-C2, H3-3A, TP53, ATRX, BRAF, KRAS, NF1, NF2, FGFR1, FGFR2, PTEN, TERT, PIK3CA, PIK3R1*), including all variants with an allele fraction ≥ 5.0%. Variants not annotated by ClinVar were in addition analysed with VarSome (www.varsome.com). As the data derives from different sources, not all data are present for all cases.

### Analyses of copy-number variation

Copy number profiles (CNP) were generated via https://www.molecularneuropathology.org. To evaluate copy-number alterations of all individual tumours, each sample was inspected visually by two different researchers. The threshold for homo- and hemizygous deletions was set individually for each case, comparing the amplitudes of different gains and losses, thus adapting the threshold to the tumour cell content. Amplifications were defined as being focal, with an amplitude larger than 0.4. For focal alterations below this threshold, we used the term “gain”.

Cumulative copy-number plots for DMGs from the clusters A and B were generated with R. The samples were normalized against publicly available control samples (CONTR_CEBM, CONTR_HEMI, CONTR_PONS, CONTR_WM), accessed via GSE109381 [7], and segmented using *conumee*. The segmented samples were cumulatively analysed using the *GenVisR* package and visualised using the *ggplot2* package. The CNV load was calculated from this data in R using the R *base* package.

### Clinical data

Parameters analysed were tumour localisation, sex, age at diagnosis and survival. For survival analysis, Kaplan–Meier survival curves were generated, and survival data was analysed further using a log-rank test in R with the packages *survival* and *survminer*. Age data was plotted with R using *ggplot2*.

### Statistics

Clinical and molecular variables were analysed with SPSS 27 (IBM, Armonk, NY, USA) and Prism 9 (GraphPad Software, Boston, MA, USA) using Chi-square statistics and logistic regression. Different localisations were compared with an ANOVA followed by a post hoc Tukey test. The level of significance was set at *p* ≤ 0.05. A forest plot based on Cox-regression was generated using the R package *survivalanalysis.*

## Results

### Unsupervised hierarchical clustering of DNA methylation data indicates two clusters of DMGs that differ in the distribution of tumour localisation and patient age

**Fig. 1:**
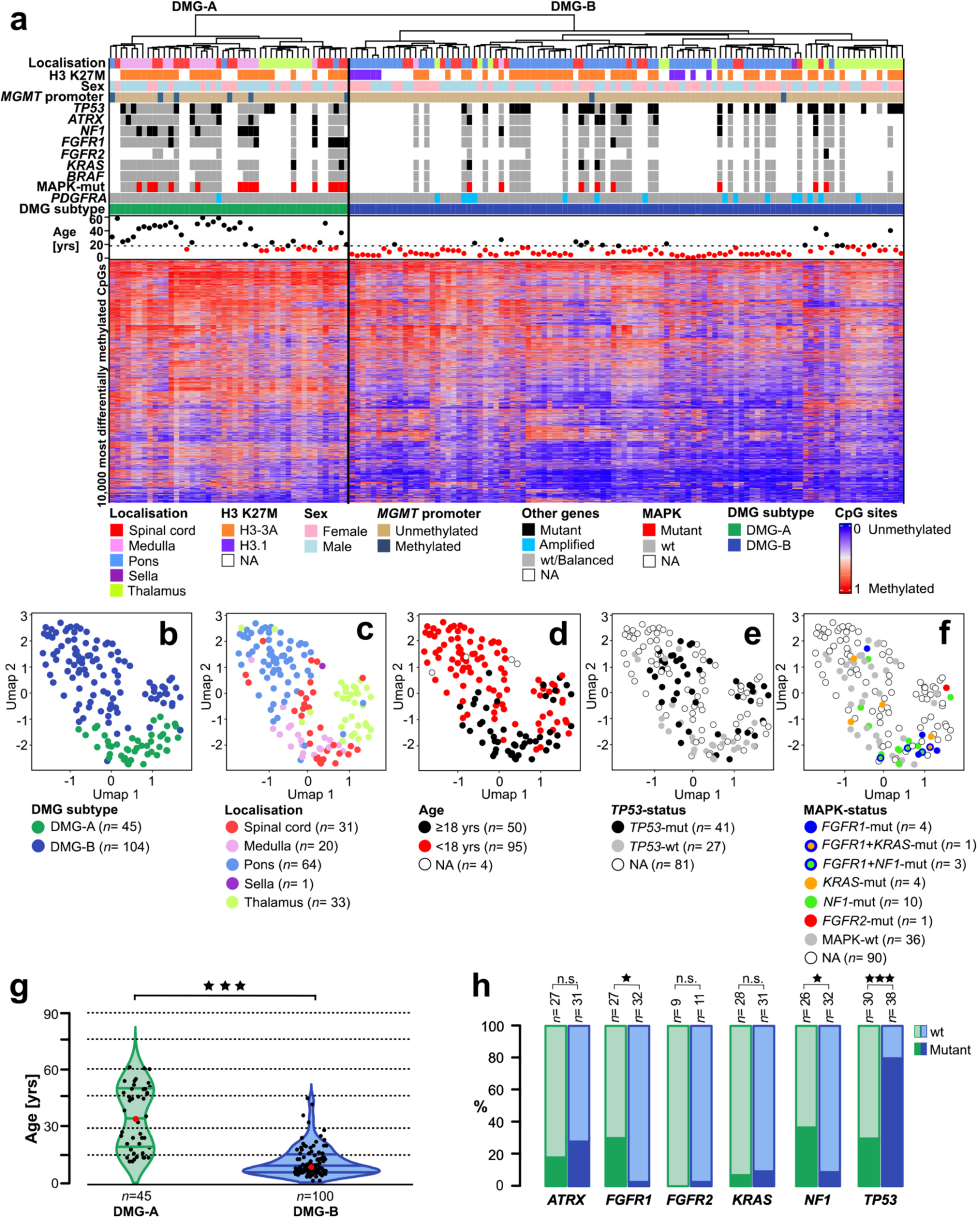
DMGs epigenetically split into two subtypes DMG-A and DMG-B that differ with respect to age, tumour localisation, *TP53*-mutations and MAPK-signalling pathway alterations. **a** Unsupervised hierarchical clustering of global DNA methylation data from 149 H3 K27M-mutant DMGs of four different localisations (spinal cord* n* = 31, medulla *n* = 20, pons *n* = 64, thalamus *n* = 33; one patient with an additional sellar tumour). DMGs subdivided into two clusters, corresponding to two DMG-subtypes DMG-A and DMG-B, that were enriched for different features. DMG-A (green, *n* = 45) was enriched for a medullary localisation (40.0%; *n* = 18 medullary cases), MAPK-associated mutations (55.6%; *n* = 15/27 cases sequenced) and cases with a methylated *MGMT* promoter (13.3%; *n* = 6/45). DMG-B (blue, *n* = 104) contained many pontine tumours (58.7%; *n* = 61) and *TP53*-mutant cases (78.9%; *n* = 30/38 cases sequenced). Most of the *FGFR1*-mutant cases formed a subcluster in the DMG-A cluster. **b**–**f** Uniform Manifold Approximation and Projection (UMAP) of the same cases shows similar results. **b** Again, DMG-A (green) and DMG-B (blue; subtype attribution from **a**) separated, as well as medullary versus pontine cases (**c**) and adult versus paediatric patients (**d**). **e** *TP53*-mutant cases were enriched in the part of the UMAP containing the DMG-B cases. **f** Most *FGFR1*- and *NF1*-mutant DMG were found in close proximity, mixed with few DMGs without known MAPK-associated alteration. **g** Violin plot of patient age. DMG-A (left) showed a bimodal age distribution with a median age at diagnosis of 31.0 ± 16.0 years. Patients with DMG-B were significantly younger (right; median age 7.6 ± 7.6 years; *p* < 0.001). The lines within the violin plots represent the quantiles (0.25, 0.50, 0.75), the red dots the median. **h** Distinct mutations were enriched in the two DMG-subtypes: *TP53*-mutations were enriched in DMG-B, while mutations associated with the MAPK-signalling pathway were enriched in DMG-A. Significance levels: *p ≤ 0.05, **p ≤ 0.01, ***p ≤ 0.001

To investigate potential epigenetic differences between DMGs from different, clearly defined localisations, we performed an unsupervised hierarchical cluster analysis of global DNA methylation data (149 DMGs in total; spinal cord *n* = 31, medulla *n* = 20, pons *n* = 64, thalamus *n* = 33, sella *n* = 1; table available as Online Resource 1). Unsupervised hierarchical clustering showed a separation of the DMGs into two main clusters. These two clusters corresponded to two subtypes of DMGs, DMG-A and DMG-B, with different clinical and molecular features that will be discussed below (Fig. 1a; DMG-A *n* = 45, DMG-B *n* = 104; average beta values in Online Resource 2). The majority of medullary cases was assigned to DMG-A (90.0%, *n* = 18/20, *p* < 0.0001). Contrarily, almost all pontine cases were assigned to DMG-B (95.3%, *n* = 61/64, *p* < 0.0001). Spinal and thalamic cases were rather evenly distributed amongst both subtypes (DMG-A: thalamus 30.3%, *n* = 10/33, *p* = 0.53; spinal cord 43.8%, *n* = 14/32, *p* = 0.17). One patient had both a spinal DMG and a second DMG in the sella. Both DMGs clustered together, belonging to DMG-B. For further statistical testing, only the spinal DMG was included.

In addition to the differences in localisation between the two subtypes, we found a significant age difference (*p* < 0.001). The median age for DMG-A was 31.0 ± 16.0 years, with a bimodal age distribution centred around adolescents and adults (25% percentile 18.0 years, 75% percentile 47.8 years, Fig. 1g). 82.2% of patients with DMG-A were adults (*n* = 34/45; medullary/spinal *n* = 29/32, pontine/thalamic *n* = 5/13), and all patients were older than 11.0 years. In contrast, DMG-B predominantly consisted of DMGs from paediatric patients, with a median age of 7.6 ± 7.6 years (*n* = 84/100 < 18 years). The enrichment of medullary cases in DMG-A (median age 43.0 ± 15.6 years.) versus pontine cases in DMG-B (6.3 ± 7.4 years) was not the only reason for the difference in patient age between the two subtypes, as spinal cases split between DMG-A and DMG-B according to age (*p* < 0.001, Fisher’s exact test). Male and female cases were equally distributed between both subtypes (sex distribution m:f: DMG-A = 1:0.6; DMG-B = 1:1.1, Fig. 1a).

We then validated the analysis with consensus clustering. k-means clustering with a pre-defined number of two clusters, as indicated by elbow and silhouette plots, gave closely resembling results, proving the robustness of the cluster analysis (Online Resource 3). Compared to the initial cluster analysis, only 5.4% of cases switched the cluster (*n* = 8/149). Next, we performed a Uniform Manifold Approximation and Reduction (UMAP) and colour-coded the cases according to the DMG-subtype (Fig. 1b). The two clusters were recapitulated in this analysis, as well as the separation of medullary versus pontine cases and paediatric versus adult cases (Fig. 1c, d).

For an additional validation of the results, we repeated the cluster analysis and UMAP with a reference cohort of 227 cases from different glioma entities and normal CNS tissue (Online Resource 4). As expected, K27M-mutant DMGs formed a cluster separate from all other entities including the *EGFR*-altered DMGs. What is more, the two DMG clusters were again present, proving the reliability of the analysis. Only 4.8% of cases switched the cluster compared to Fig. 1a (*n* = 5/104).

This data indicates that medullary and pontine DMGs are epigenetically dissimilar, resulting in an assignment of the medullary cases to DMG-A and of the pontine cases to DMG-B. Spinal and thalamic DMGs are epigenetically more diverse and scatter across both DMG-subtypes, partially according to age. DMG-A showed a bimodal age distribution, arising mainly in adolescents and adults, whilst DMG-B was mainly detected in paediatric patients.

### DMG-A has significantly more mutations associated with the MAPK-signalling pathway whereas DMG-B has more *TP53*-mutations

Next, we analysed the *MGMT* promoter methylation status and checked for mutations in a subset of DMGs.

13.3% (*n* = 6/45) of DMG-A had a methylated *MGMT* promoter as opposed to only 1.9% (*n* = 2/104) of DMG-B (*p* < 0.01; Fig. 1a). Half of the DMGs with a methylated *MGMT* promoter were from the spinal cord and about one third from the pons. DMGs from the spinal cord also showed the highest percentage of cases with a methylated *MGMT* promoter, whilst thalamic DMG never had a methylated *MGMT* promoter (spinal cord: *n* = 4/31, 12.9%, medulla: *n* = 1/20, 5.0%, pons: *n* = 3/64, 4.1%, thalamus: *n* = 0/33, 0.0%). 75% of DMGs with a methylated *MGMT*-promoter derived from adults (*n* = 6/8).

We subsequently analysed the CNP of DMGs for amplifications. The most frequent alteration, present in 16 DMGs, was an amplification of the platelet-derived growth factor alpha (*PDGFRA*), which also plays a role in tumour cell proliferation and migration [11, 16]. Amplifications of *PDGFRA* were exclusively found in *H3.3*-mutant DMGs, and significantly more often in DMG-B (*p* = 0.04; DMG-B 14.4%, *n* = 15/104; DMG-A 2.2%, *n* = 1/45; Fig. 1a, Online Resource 1). In addition, 2.2% (*n* = 1/45) of DMG-A and 2% (*n* = 3/104) of DMG-B showed a gain of *PDGFRA* (amplitude < 0.4). The second amplification that occurred in both subtypes was an amplification of *CCND1* (DMG-A 2.2%, *n* = 1/45; DMG-B 2.9%, *n* = 3/104). Amplifications of *EGFR, MDM2, CCND2, CDK4* and *MET* occurred in single cases only (Online Resource 1).

The different frequency of *PDGFRA* gains and amplifications was also visible in cumulative copy-number plots of DMG-A and DMG-B (Online Resource 5). In general, gains and losses were present in similar chromosomal regions in CNP from both DMG subtypes. However, DMG-B had significantly more copy-number alterations, especially losses, than DMG-A (*p* < 0.001; mean CNV load/Mb DMG-A 183.5 ± 168.5, DMG-B 258.1 ± 127.0).

We then analysed the distribution of different H3-mutations, *TP53-, ATRX-* and MAPK-related mutations (*NF1, FGFR1*, *FGFR2, KRAS* and *BRAF*) in the two subtypes. Sequencing data was available for 94 cases (spinal cord *n* = 11, medulla *n* = 20, pons *n* = 50, thalamus *n* = 13, DMG-A: *n* = 30, DMG-B: *n* = 64). The vast majority of the samples harboured an H3-3A K27M mutation (88.3%, *n* = 83/94; Fig. 1a). The samples with an H3.1 K27M mutation formed clusters separate from the cases with H3.3 K27M, exclusively within the DMG-B cluster (Fig. 1a and Online Resources 3c**,** 4a). All cases with an H3.1 K27M mutation originated from the pons, which is in line with the literature [9].

Besides the H3 K27M mutation, DMGs frequently harboured mutations in the tumour suppressor genes *TP53* (60.3%, *n* = 41/68), *NF1* (22.4%, *n* = 13/58), *ATRX* (19.0%, *n* = 11/58) and *PTEN* (8.9%, *n* = 5/56), as well as in the proto-oncogenes *FGFR1* (13.6%, *n* = 8/59), *FGFR2* (5.0%, *n* = 1/20) and *KRAS* (8.6%, *n* = 5/58) (Online Resource 1)*.*

Of note, mutations in genes associated with the MAPK signalling pathway (*NF1, FGFR1*, *FGFR2* and *KRAS*) were significantly enriched in DMG-A: 55.6% of DMG-A had a MAPK-related mutation (*n* = 15/27), as opposed to only 25.0% of DMG-B (*n* = 8/32; *p* = 0.031; Fig. 1h, Online Resource 1). Mutations in *NF1* and *FGFR1* were predominant*,* whereas *BRAF-*mutations were not present. *NF1*-mutations were the most frequent MAPK-associated mutations, present in 38.5% of DMG-A (*n* = 10/26, *p* = 0.012; Fig. 1h). On the contrary, only 9.4% of DMG-B were *NF1*-mutant (*n* = 3/32). DMG-A also contained 25.9% *FGFR1*-mutant cases (*n* = 7/27), as opposed to only 3.1% *FGFR1*-mutant DMG-B (*n* = 1/32; *p* = 0.019; Fig. 1h). Of note, most *FGFR1*-mutant cases clustered together in one subcluster (Fig. 1a and Online Resources 3c, 4a). Still, we did not find a clear separation of *FGFR1*-mutant cases from all other cases in the UMAP, as described by Auffret and colleagues [1] (Fig. 1f). *FGFR1*-mutations were present in DMGs of all localisations, except the thalamus, and across all age groups. Conversely, more than two thirds of the *NF1*-mutant cases were detected in tumours from adult patients, predominantly in medullary localisation. This shows that some mutations occur in certain tumour localisations or related with a certain age at diagnosis, whilst other alterations are more universally found. Of note, 86.0% of all MAPK-mutations occurred in patients ≥ 10 years, i.e. in adolescents and adults.

Previous studies have described the coexistence of an *NF1*-mutation with further alterations in the MAPK-signalling pathway [29]. In our cohort, three medullary cases had mutations in both *FGFR1* and *NF1* and one spinal case had mutations in *FGFR1* and *KRAS* (Fig. 1a, f; Online Resource 1). Hence, half of the *FGFR1*-mutant cases (*n* = 4/8) had an additional MAPK-related mutation.

Opposingly, *TP53*-mutations were significantly enriched in DMG-B (*p* < 0.001): 78.9% (*n* = 30/38) of DMG-B were *TP53*-mutant as opposed to only 36.6% (*n* = 11/30) of DMG-A (Fig. 1h). Prominent was the significantly higher percentage of *TP53* mutations in pontine and thalamic DMGs, compared to spinal and medullary DMGs (spinal cord: *n* = 3/9, 33.3%, medulla: *n* = 5/20, 15.0%, pons: *n* = 20/25, 80.0%, thalamus: *n* = 11/13, 84.6%; *p* < 0.05), which is in line with the literature [12]. The percentage of *ATRX*-mutant cases was very similar in both subtypes (DMG-A: 18.5%, *n* = 5/27; DMG-B: 19.4%, *n* = 6/31; *p* > 0.99; Fig. 1h).

A logistic regression showed that the odds for cases with a MAPK-associated mutation to be included in the DMG-A cluster were significantly higher than to be included in the DMG-B cluster (*p* = 0.019, Odds ratio (OR) 3.75), whilst for *TP53*-mutant cases the opposite was true (*p* < 0.001, (OR) 0.113).

In summary, we find that DMG-A contains significantly more cases with a methylated *MGMT* promoter and mutations associated with the MAPK-signalling pathway, and DMG-B significantly more *PDGFRA*-amplifications and *TP53*-mutations.

### The prognosis of DMG-A is significantly better than of DMG-B

**Fig. 2:**
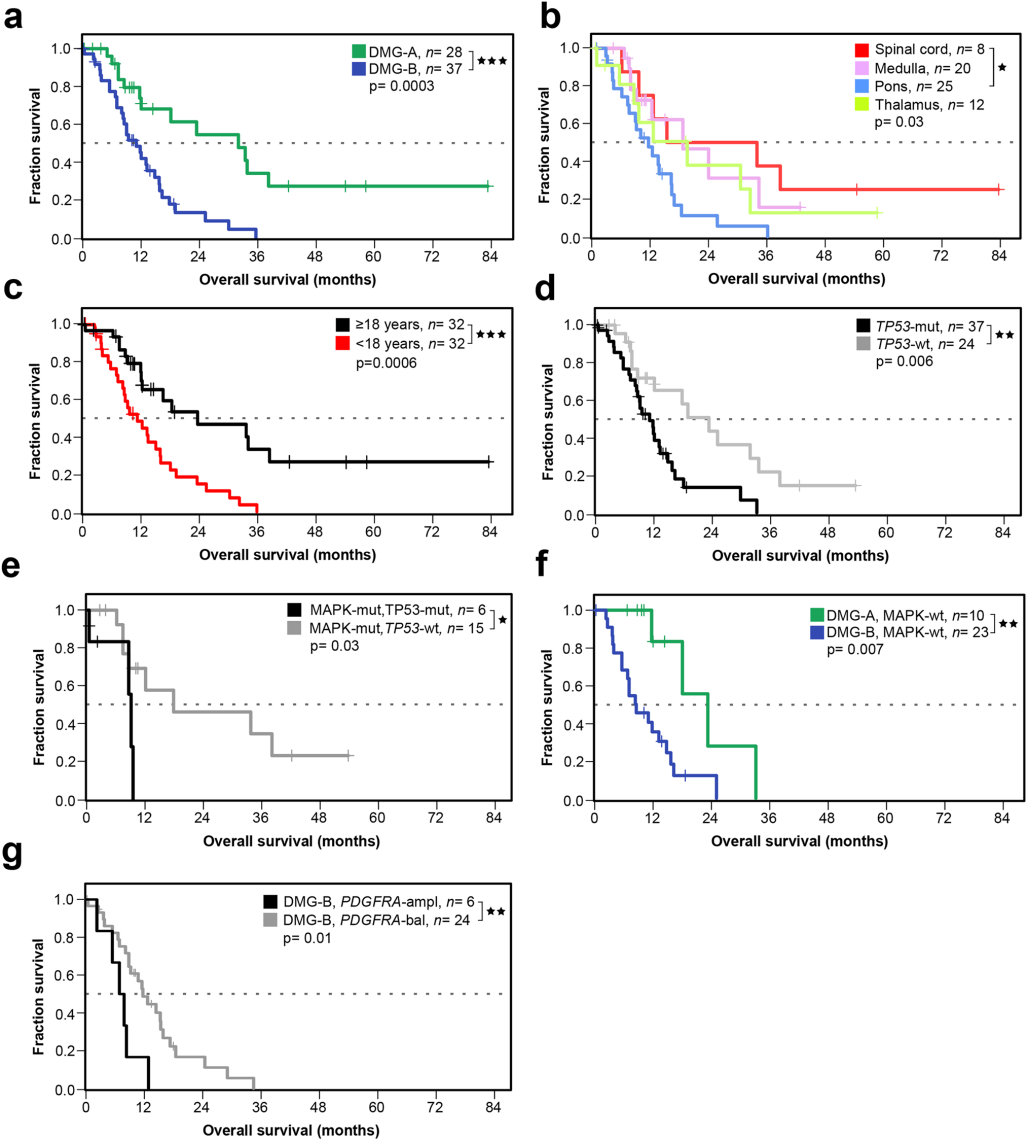
Patients with DMG-A have a significantly better survival than patients with DMG-B. Overall survival (OS) for all cases with survival data available (*n* = 65; spinal cord: *n* = 8, medulla: *n* = 20, pons: *n* = 25, thalamus: *n* = 12). **a** Patients with DMG-A have a significantly better survival than patients with DMG-B (DMG-A: median OS = 32.0 ± 20.0 months; DMG-B: 11.0 ± 10.3 months, *p* < 0.001). In univariate analyses, OS also differed for spinal versus pontine DMGs (**b**; spinal cord: median OS = 24.1 ± 26.9 months, medulla: median OS = 18.1 ± 9.9 months, pons 11.0 ± 11.0 months, thalamus: median OS = 19.0 ± 17.0 months), for children versus adults (**c**; < 18 years: median OS = 11.0 ± 8.9 months, ≥ 18 years: 23.4 ± 19.0 months), for patients with *TP53*-mutant versus *TP53*-wild type (wt) DMGs (**d**; *TP53*-mutant: median OS = 11.0 ± 7.3 months, *TP53*-wild type: 23.4 ± 13.9 months), for DMGs that were MAPK-mutant, *TP53*-wild type versus MAPK-mutant, *TP53*-mutant (**e**, MAPK-mut/*TP53*-wt: OS = 17.8 ± 16.2 months and MAPK-mut/*TP53*-mut: OS = 9.0 ± 4.6 months), for DMG-A, MAPK wild type versus DMG-B, MAPK wild type (**f**, DMG-A, MAPK-wt: OS = 23.4 ± 8.2 months and DMG-B, MAPK-wt: OS = 8.4 ± 6.8 months) and DMG-B without and with *PDGFRA*-amplification (**g**, DMG-B, *PDGFRA*-balanced: OS = 12.0 ± 8.3 months; DMG-B, *PDGFRA*-amplified: OS = 7.5 ± 3.4 months). However, tumour localisation, *TP53*-status, *PDGFRA*-status and age were not independent of the cluster attribution. Significance levels: *p ≤ 0.05, **p ≤ 0.01, ***p ≤ 0.001

Next, we analysed the OS for all cases with available data (*n* = 65; spinal cord: *n* = 8, medulla: *n* = 20, pons: *n* = 25, thalamus: *n* = 12). OS for patients with DMG-A was significantly better compared to DMG-B (Fig. 2a; *p* < 0.001; DMG-A: median OS = 32.0 ± 20.0 months; DMG-B: median OS = 11.0 ± 10.3 months).

We also detected significant differences in OS associated with further parameters, which, as discussed below, were not independent of the cluster attribution: Compared to patients with pontine DMGs, both the survival of patients with spinal DMGs and all non-pontine DMGs combined were significantly better (Fig. 2b; spinal cord: median OS = 24.1 ± 26.9 months, medulla: median OS = 18.1 ± 9.9 months, pons: median OS = 11.0 ± 11.0 months, thalamus: median OS = 19.0 ± 17.0 months; *p* = 0.03 resp. 0.006). However, amongst DMG-B, the survival of patients with DMG-B of non-pontine localisations was as poor as the survival of patients with pontine DMG-B (DMG-B, non-pontine: median OS = 10.5 ± 8.1 months, DMG-B, pons: median OS = 11.0 ± 7.8 months, *p* = 0.7, Online Resource 6d). This confirms that patients with DMG-B have a worse prognosis compared to DMG-A, independent of the localisation.

Adults had a better OS than children (Fig. 2c; < 18 years: median OS = 11.0 ± 8.9 months, ≥ 18 years: 23.4 ± 19.0 months, *p* = 0.0006). As published previously [32], the OS of patients with *TP53*-wild type (wt) DMGs was significantly better than that of patients with *TP53*-mutant DMGs (Fig. 2d; *TP53*-mutant: median OS = 11.0 ± 7.3 months, *TP53*-wild type: median OS = 23.4 ± 13.9 months, *p* = 0.006). We then combined these two factors for further analyses. Only two DMG-A, *TP53*-mutant were derived from paediatric patients, as opposed to 20 *TP53*-mutant DMG-B. Amongst paediatric patients with *TP53*-mutant DMG-B, the survival of patients with non-pontine DMG-B was as poor as the survival of those with pontine DMG-B (DMG-B, *TP53*-mutant, non-pontine, < 18 years: median OS = 12.0 ± 8.9 months, DMG-B, *TP53*-mutant, pons, < 18 years: median OS = 8.3 ± 4.5 months, *p* = 0.2, Online Resource 6**e**). This shows that DMG-B integrates different parameters associated with a poor prognosis, irrespective of the tumour localisation.

MAPK-mutant, *TP53*-wild type cases had a significantly better survival than MAPK-mutant, *TP53*-mutant cases (Fig. 2e; MAPK-mut/*TP53*-wt: median OS = 17.8 ± 16.2 months and MAPK-mut/*TP53*-mut: median OS = 9.0 ± 4.6 months; *p* = 0.03). The MAPK-status alone did not significantly influence survival (MAPK-mutant: median OS = 12.0 ± 15.3 months, MAPK-wt: median OS = 11.8 ± 7.4 months, *p* = 0.2, Online Resource 6**a**). This was also not the case when looking at individual MAPK-alterations (*FGFR1*-mut: median OS = 17.9 ± 28.3 months, *NF1*-mut: median OS = 33.8 ± 25.0 months, *KRAS*-mut: median OS = 9.0 ± 13.9 months; *p* = 0.3, Online Resource 6**b**). However, DMG-A, MAPK-wt cases had a significantly better survival than DMG-B, MAPK-wt cases (Fig. 2f; DMG-A, MAPK-wt: OS = 23.4 ± 8.2 months and DMG-B, MAPK-wt: OS = 8.4 ± 6.8 months; *p* = 0.007). The prognosis of patients with DMGs harbouring a *PDGFRA*-amplification was in general worse than that of patients with DMGs having a balanced *PDGFRA*-locus (*p* < 0.001). As 88% of *PDGFRA*-amplified cases with available sequencing data were also *TP53*-mutant (n = 7/8), we also tested the survival of patients with DMG-B harbouring a *PDGFRA*-amplification versus DMG-B with a balanced *PDGFRA*-locus, and again detected a significant difference (*p* = 0.01; Fig. 2g; DMG-B, *PDGFRA*-balanced: OS = 12.0 ± 8.3 months; DMG-B, *PDGFRA*-amplified: OS = 7.5 ± 3.4 months). A difference in the OS between male and female patients was not detected (Online Resource 6c; female: median OS = 13.0 ± 17.5 months, male: median OS = 14.8 ± 12.1 months, *p* = 0.2).

Summarising these findings, individual features primarily detected in DMG-B (pontine localisation, *TP53*-mutant, *PDGFRA*-amplified, paediatric patients) were associated with a poorer prognosis, compared to features enriched in DMG-A. In line with this, a univariate cox regression also identified subtype, tumour localisation, *PDGFRA* copy-number status, *TP53*-status and age as significant variables (Online Resource 6f). Fisher’s Exact tests proved that tumour localisation, *PDGFRA*-status, *TP53*-status and age at diagnosis were dependent on the DMG-subtype (*p* < 0.05 for all tests). In agreement, a multivariate Cox-regression did not indicate any significant parameter.

**Fig. 3:**
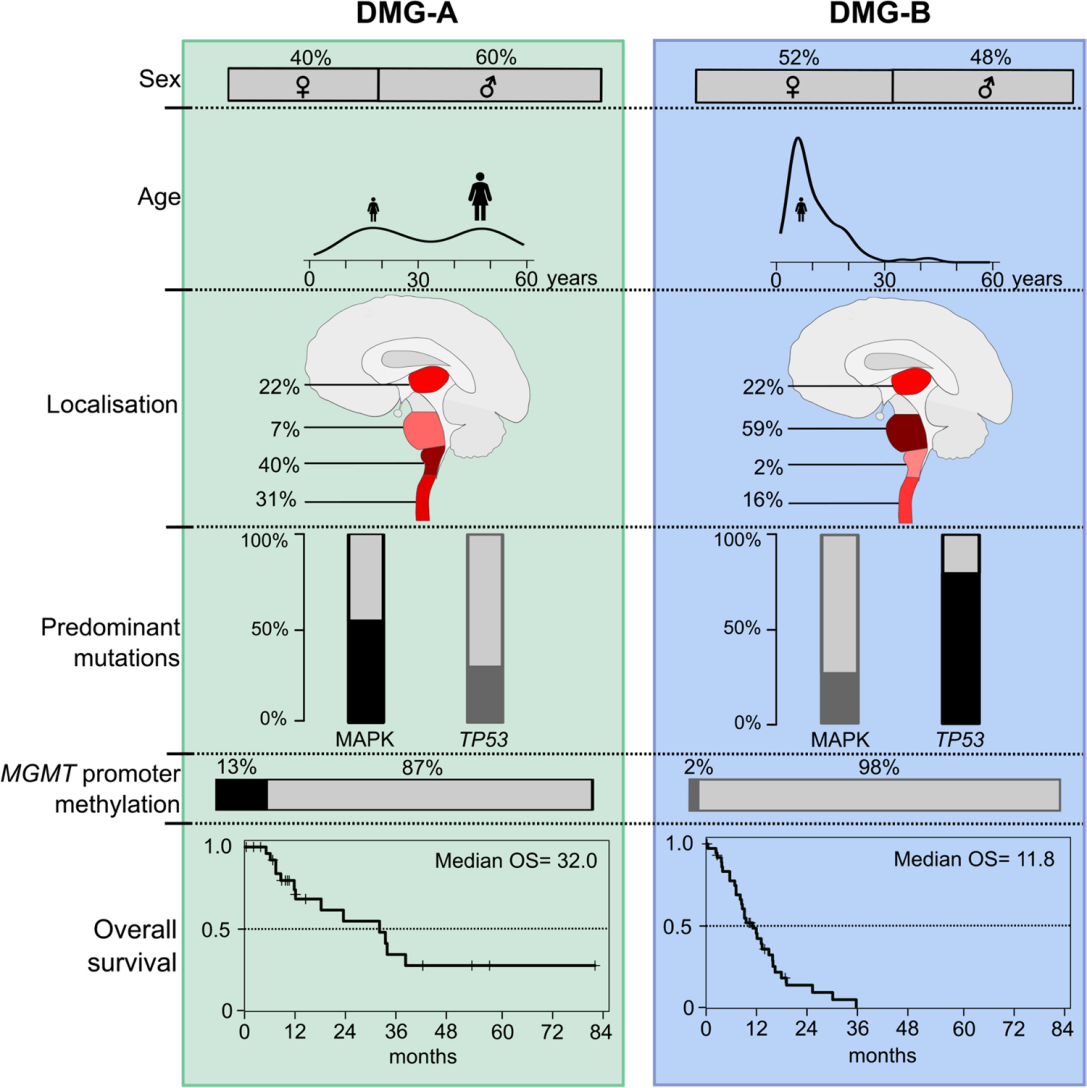
Summary of the clinical and molecular features of DMG-A and DMG-B. DMG-A is enriched for adult patients, medullary localisation and MAPK-associated mutations*,* and contains more cases with a methylated *MGMT* promoter. Contrarily, DMG-B is enriched for paediatric patients, pontine localisation and cases with *TP53*-mutations. The overall survival of patients with DMG-A is superior to that of patients with DMG-B

In summary, an unbiased stratification via unsupervised hierarchical clustering is well suited to predict OS as it integrates different parameters that influence survival. Hence, a stratification according to the two DMG-subtypes A and B showed the most significant difference in survival. The features of DMG-A and DMG-B are summarised in Fig. 3.

## Discussion

The aim of the project was to find clinically meaningful subtypes of DMG, integrating epigenetic, sequencing and clinical data in a large cohort of DMGs from different, clearly defined localisations. So far, studies focussed on either localisation, the specific H3-mutation or mutations associated with the MAPK-signalling pathway, and long- versus short-term survival [1, 12, 32]. Here, we investigated how these parameters collectively influence survival.

We analysed tumours from four clearly defined localisations without prior knowledge of the mutational status and survival status (long- versus short-term survival). Thus, our cohort is heterogeneous, representing the different facets of DMG biology. In several studies [1, 32, 33], medullary and pontine tumours were combined as “brainstem” localisation, while we separated tumours from these localisations. Hence, a direct comparison of our results to the findings from these studies is not feasible. Our rationale for a separation of medullary and pontine DMGs was that they have very different mutations and different clinical features: medullary DMGs mainly occur in adults and often have MAPK-associated mutations while pontine DMGs mainly occur in children and are enriched for *TP53*-mutations. The differences between medullary and pontine DMGs are also reflected in a different epigenetic profile, as already shown by Chen and colleagues, who exclusively studied pontine and medullary DMGs [12].

We find that the DMGs robustly subdivide into two methylation clusters. These clusters correspond to two subtypes DMG-A and DMG-B with different clinical and molecular features: DMG-A was enriched for adult patients, medullary localisation, and MAPK-associated mutations, and contained significantly more DMGs with a methylated *MGMT*-promoter. On the contrary, DMG-B mainly contained tumours from paediatric patients, predominantly in pontine localisation, and was enriched for *TP53*-mutations, and, to a lower extent, *PDGFRA*-amplifications. This also shows that the features of DMGs from paediatric and adult patients differ, both with regard to the tumour localisation as well as regarding mutations and the epigenetic profiles of the tumours. Spinal and thalamic cases were found in both clusters. This again indicates that the tumour localisation is not the only parameter influencing the epigenetic profile of DMGs but also parameters such as the mutational spectrum and patient age.

Several features that went along with a poorer survival in univariate analyses, such as pontine localisation, *TP53*-mutations and paediatric age, were enriched in DMG-B. Therefore, it is no surprise that these characteristics are neither independent of each other, nor of the cluster attribution. Consequently, the DMG-subtypes corresponded with the strongest survival difference: patients with DMG-A had a better survival than those with DMG-B. Hence, a classification of DMG-subgroups according to methylation clustering is well suited to predict survival as it integrates different molecular and clinical parameter that will be discussed below.

We detected many differences between DMG-A and DMG-B. In previous studies, DMGs rarely had a methylated *MGMT* promoter [2, 23]. Since the standard treatment for H3 K27M-altered DMGs includes TMZ, a chemotherapeutic agent whose efficacy is decreased if the *MGMT* promoter is unmethylated, the methylation status of the *MGMT* promoter is crucial for the response to the treatment. In our cohort, there was a significant difference of cases with a methylated *MGMT* promoter between the two subtypes: 13.3% of DMGs from DMG-A had a methylated *MGMT* promoter, as opposed to only 1.9% of the DMGs from DMG-B. This hints at biological differences of the cases belonging to the two subtypes, and indicates that special attention needs to be given to the *MGMT* promoter methylation status of DMGs attributed to DMG-A. The percentage of 13.3.% of cases with a methylated *MGMT* promoter is much higher than the highest percentage of 4.5% detected in a DMG cohort so far [26], and it often occurred in adults and spinal DMGs. Whilst it is likely that these patients may profit considerably from the treatment with TMZ, unfortunately, survival data was only available for four DMGs with a methylated *MGMT* promoter in our cohort. Thus, a follow up study may analyse the survival of DMGs with a methylated *MGMT* promoter.

Both an inactivation of *TP53* and *PDGFRA*-amplifications have been associated with a poor OS in DMGs [10, 13], which we could confirm with our data. However, *TP53*-mutations were more frequent in pontine and thalamic DMGs as compared to spinal and medullary DMGs, and the *TP53*-status was neither independent of the tumour localisation nor of the subtype attribution. The same is true for the *PDGFRA*-amplification. Hence, different parameters are interconnected in the two subtypes of DMGs. Previous studies revealed that mutations in *TP53* are often associated with *FGFR1* wild type status and vice versa [33]. Consistently, in our cohort, only one *TP53-*mutant sample also had an *FGFR1*-mutation (*n* = 1/41, 2.4%).

Looking at mutations in genes associated with the MAPK-signalling pathway in general, more than a third of the samples presented with one or several of the MAPK mutations analysed, which is in line with the literature [1, 29, 32]. MAPK-related alterations represent potential therapeutic targets which need to be taken into close consideration for a combination therapy [14, 22]. *FGFR1*-mutations rarely occur in children [1, 32], but in DMGs of adolescent and adult patients. As opposed to previous studies [1, 32, 33], we did not find that DMGs with MAPK-associated mutations have a better survival than those without MAPK-associated mutations. The DMGs analysed in our study did not harbour *BRAF*-mutations which may influence survival. However, most likely the difference between our study and previous studies results from a different selection of cases. Roberts and colleagues [32] took a different starting point, specifically collecting data from long-term survivors. They could then associate MAPK-alterations with long-term survival. In our cohort, only few patients having DMGs with MAPK-mutations were long-term survivors (LTS). If defining LTS as survival > 36 months and short-term survival (STS) < 18 months as done by Roberts and colleagues [32], 81.0% (*n* = 17/21) of MAPK-altered tumours were STS while only three patients were LTS (median OS of all MAPK-altered DMG 12.0 months). However, half of the patients with MAPK-altered DMGs were still alive at the latest follow-up. Amongst the eight *FGFR1*-mutant cases, seven were STS and only one a LTS. Likely the reason for this is that in addition, different factors such as *TP53*-status and tumour localisation influence survival. For instance, in our cohort, MAPK-altered DMGs that were *TP53*-wild type had a significantly better survival compared to MAPK-altered DMGs that were *TP53*-mutant. In the cohort of Auffret et al. [1], the median OS of patients with *FGFR1*-mutant DMGs was 36 months, and the same OS was detected for all MAPK-mutant DMGs in the study of Roberts and colleagues [32]. This OS of 36 months for MAPK-mutant DMGs in both studies is very similar to the median OS of 32 months for the DMG-A subtype in our study. As 44% of DMG-A in our cohort with MAPK-status do not have a MAPK-alteration, this again illustrates that different parameters, not only the MAPK-status, inseparably influence survival.

In sum, our data show that different factors such as age, localisation and *TP53* status influence survival of patients with DMG. Of note, these different factors are not independent. Hence, looking only at one of these factors is not sufficient to stratify survival of DMGs. Clustering of DNA methylation data allows to integrate these factors as DMG are separated into two clusters that are enriched for differing features, corresponding to two subtypes of DMG. Most important, survival of patients with DMGs from these two subtypes differs significantly, and the effect was larger than that of all other parameters tested. Therefore, we propose a methylation-based classification of two subtypes of DMGs that can be used to predict survival as it integrates different molecular and clinical parameters. This model may be especially useful for patients with non-pontine tumours: Whilst many non-pontine tumours are DMG-A and patients have a good prognosis, the classification may be used to detect patients with non-pontine DMG-B that may fare much worse.

## Supplementary Information

Below is the link to the electronic supplementary material.

Supplementary file1 (XLSX 1224 KB)

Supplementary file2 (XLSX 16545 KB)

Supplementary file3 (PDF 6332 KB)

## Data Availability

The methylation data from DMGs processed in Hamburg (*n* = 28) have been deposited at Gene Expression Omnibus under the accession GSE270993. The average beta values of the 10,000 most differentially methylated CpG sites of all 149 DMGs are available as Online Resource 2.
