# Supplementary material for: Integrated analyses reveal two molecularly and clinically distinct subtypes of H3 K27M-mutant diffuse midline gliomas with prognostic significance
Source: Acta Neuropathol. 2024 Sep 10;148(1):40. doi: 10.1007/s00401-024-02800-3 (PMC11387453; doi:10.1007/s00401-024-02800-3)
Supplement: Supplementary file 3 — Supplementary file3 (PDF 6332 KB) [file 401_2024_2800_MOESM3_ESM.pdf]

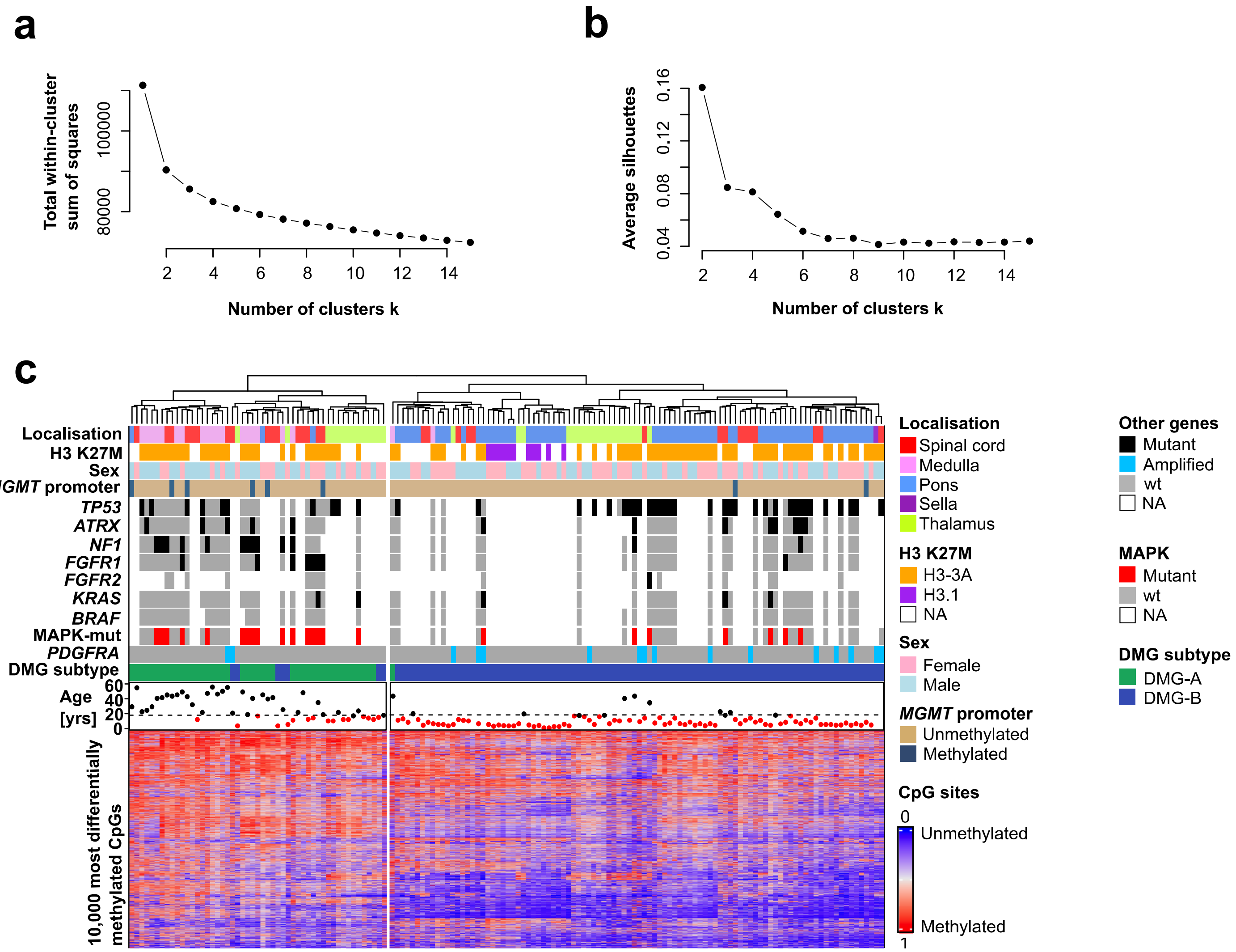

Online Resource 3: k-means clustering confirms the two DMG subtypes. Consensus clustering of 149 H3 K27M-mutant DMGs (**c**) confirms the presence of two different subtypes of DMGs, DMG-A and DMG-B. The optimal number of two clusters was determined using the elbow method (**a**) and the silhouette method (**b**), and proved the robustness of the number of two main clusters from Fig. 1a. Only minor differences were visible: eight cases switched the cluster compared to the unsupervised hierarchical cluster analysis from Fig. 1 (DMG-A green, DMG-B blue). Otherwise, results were comparable. Most *FGFR1*-mutant cases clustered together in one subcluster of DMG-A cases, while H3.1 cases clustered together in neighbouring subclusters of DMG-B.

"Integrated analyses reveal two molecularly and clinically distinct subtypes of H3 K27M-mutant diffuse midline gliomas with prognostic significance"

Acta Neuropathologica, Stegat L et al.
Corresponding author: Dr. Annika K. Wefers, Institute of Neuropathology, University Medical Center Hamburg-Eppendorf, Hamburg, Germany; present address: Department of Neuropathology, Regensburg University Hospital, Regensburg, Germany, annika.wefers@ukr.de

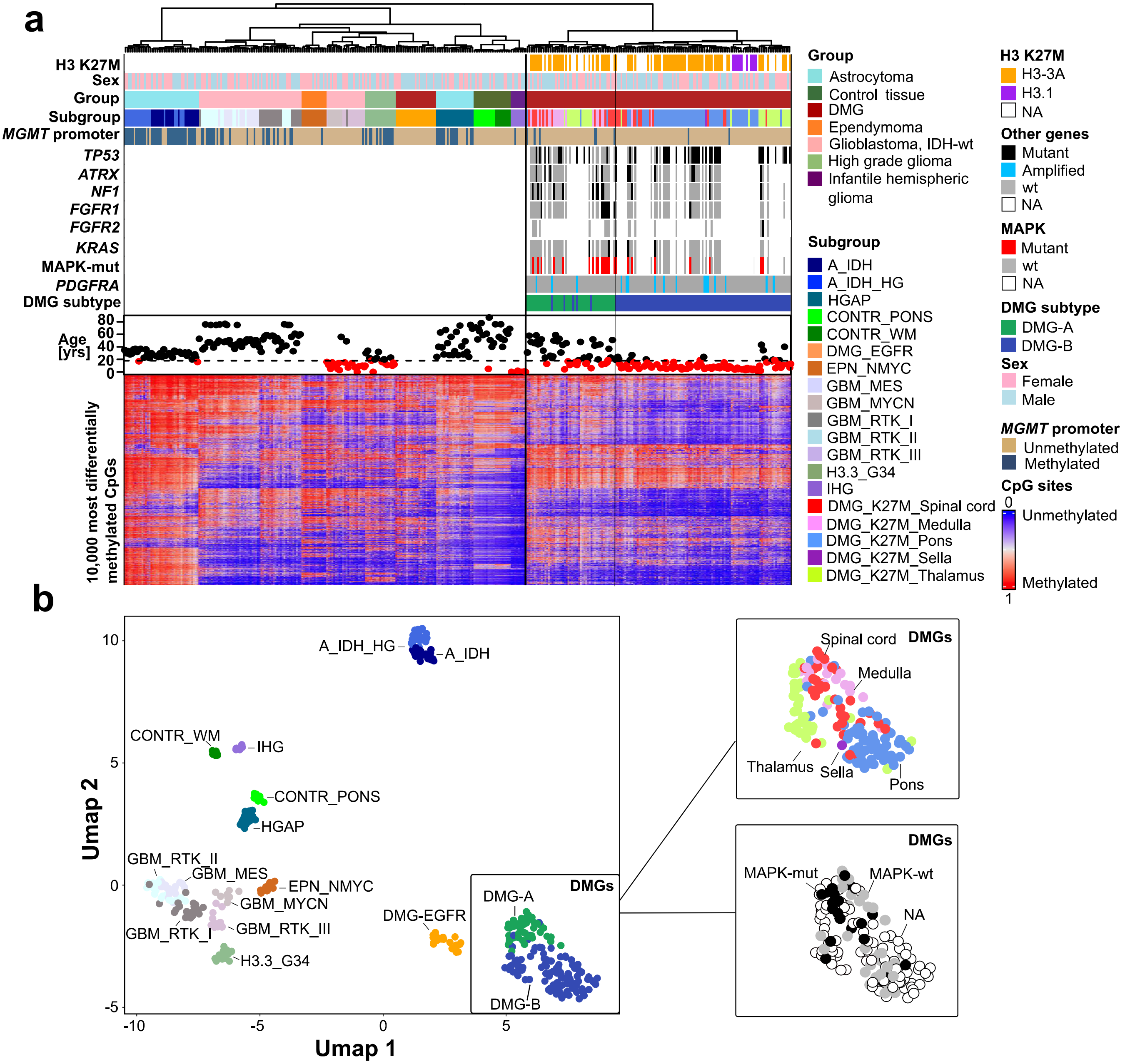

Online Resource 4: Unsupervised hierarchical clustering and Uniform Manifold Approximation and Projection (UMAP) for Dimension Reduction of DMGs with different reference classes prove the robustness of the results
Unsupervised hierarchical clustering (**a**) and UMAP (**b**) of the DMGs with a reference cohort of 227 cases from different glioma entities and normal CNS tissue confirm the two DMG-subtypes from Fig. 1a. In **a,** the cases from DMG-A in Fig. 1a are shown in green, those from DMG-B in blue. Only five cases switched the cluster. Again, the DMG-A cluster contained DMGs with more medullary DMGs (**a,** see also upper right panel in **b**) and more mutations in genes associated with the MAPK-signalling pathway (**a,** see also lower right panel in **b**). The DMG-B cluster contained more pontine DMGs (**a** and upper right panel in **b**) and *TP53*-mutant cases (**a**). Reference methylation classes: Astrocytoma, *IDH*-mutant (A_IDH); High grade astrocytoma *IDH*-mutant (A_IDH_HG); High-grade astrocytoma with piloid features (HGAP); Control tissue, pons (CONTR_PONS); Control tissue, white matter (CONTR_WM); Diffuse midline glioma, *EGFR*-altered (DMG_EGFR); Ependymoma, subclass NMYC (EPN_NMYC); Glioblastoma, *IDH*-wildtype, subclass mesenchymal (GBM_MES); Glioblastoma, *IDH*-wildtype, subclass *MYCN* (GBM_MYCN); Glioblastoma *IDH*-wildtype, subclass RTK I (GBM_RTK_ I), Glioblastoma *IDH*-wildtype, subclass RTK II (GBM_RTK_II), Glioblastoma *IDH*-wildtype, subclass RTK III (GBM_RTK_III); Diffuse hemispheric glioma, H3 G34-mutant (H3.3_G34); Infant-type hemispheric glioma (IHG)

“Integrated analyses reveal two molecularly and clinically distinct subtypes of H3 K27M-mutant diffuse midline gliomas with prognostic significance"
Acta Neuropathologica, Stegat L et al.
Corresponding author: Dr. Annika K. Wefers, Institute of Neuropathology, University Medical Center Hamburg-Eppendorf, Hamburg, Germany; present address: Department of Neuropathology, Regensburg University Hospital, Regensburg, Germany,annika.wefers@ukr.de

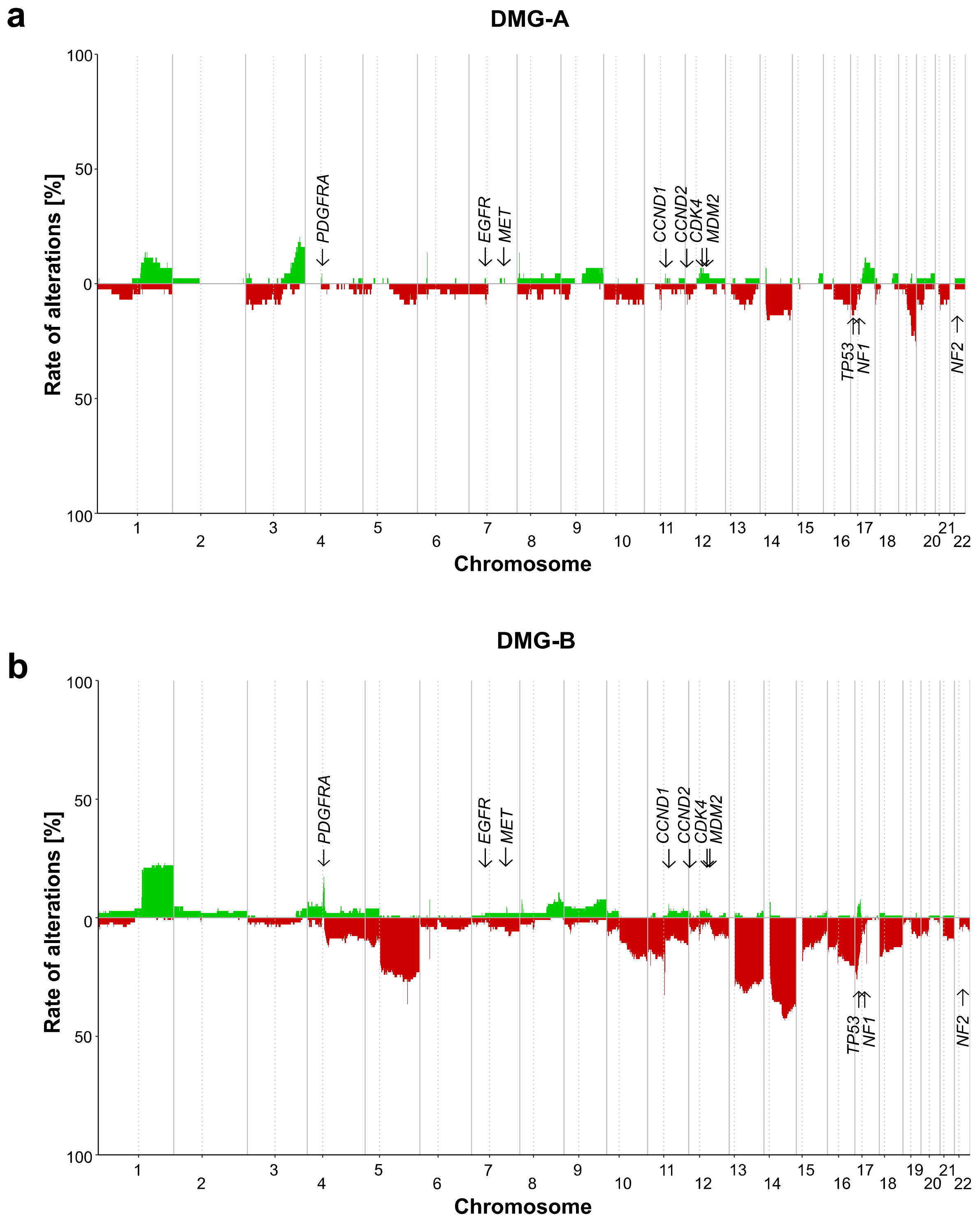

Online Resource 5: DMG-B have significantly more copy number alterations than DMG-A Cumulative copy number plots of cases from DMG-A (**a**) and DMG-B (**b**). Rate of gains in green, rate of losses in red. Arrows at the top mark the loci of selected proto-oncogenes that were amplified in some DMGs. Arrows at the bottom mark the loci of selected tumour suppressors that were deleted in some DMGs. Mean CNV load/Mb: DMG-A 183.5 ± 168.5, DMG-B 258.1 ± 127.0 ($p < 0.001$)

"Integrated analyses reveal two molecularly and clinically distinct subtypes of H3 K27M-mutant diffuse midline gliomas with prognostic significance"

Acta Neuropathologica, Stegat L et al.

Corresponding author: Dr. Annika K. Wefers, Institute of Neuropathology, University Medical Center Hamburg-Eppendorf, Hamburg, Germany; present address: Department of Neuropathology, Regensburg University Hospital, Regensburg, Germany, annika.wefers@ukr.de

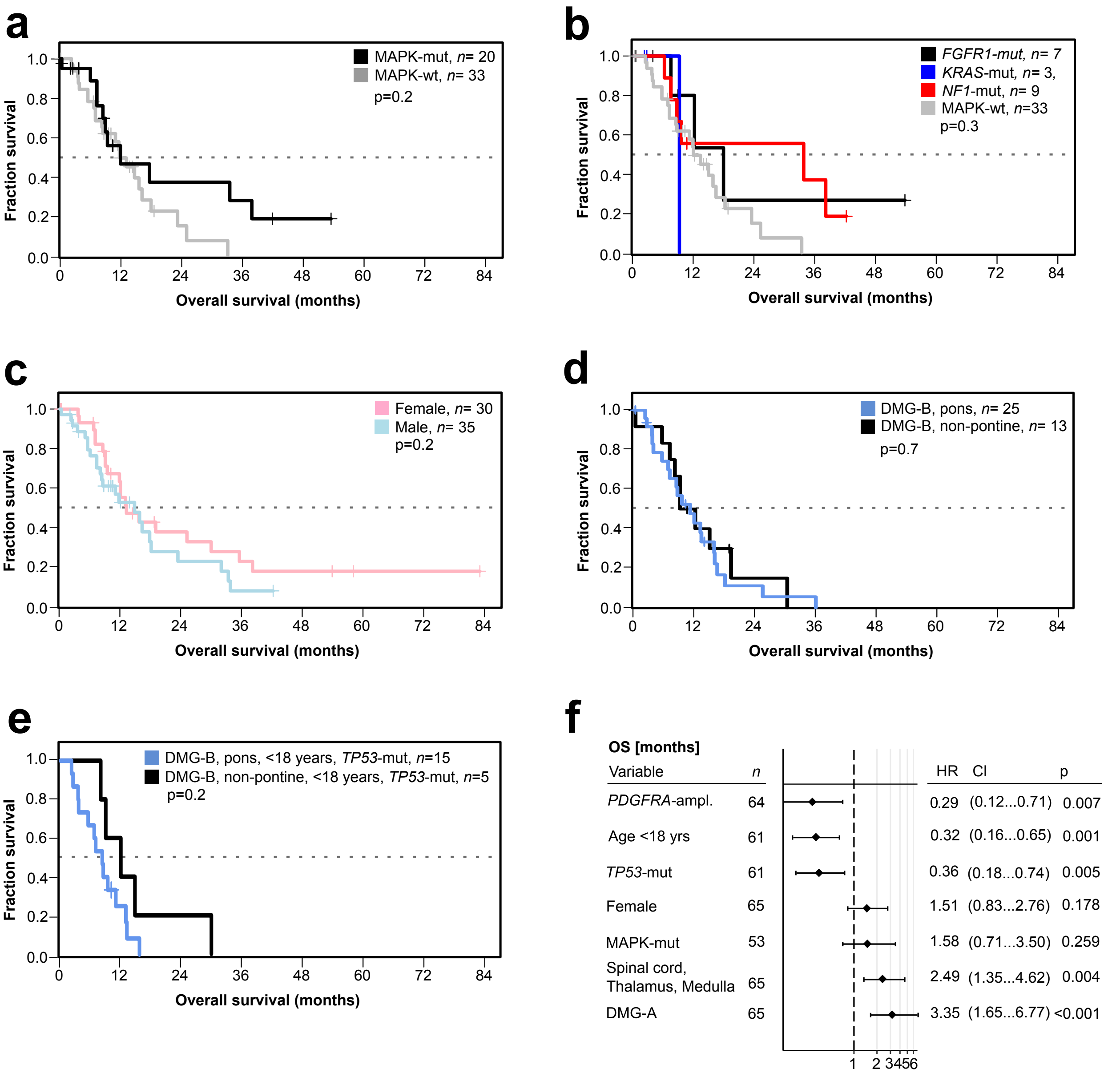

Online Resource 6: **MAPK-status and sex do not influence survival**. MAPK-status in general (**a**), as well as individual MAPK-mutations (**b**) and sex (**c**) did not influence survival (MAPK-mutant: median OS= 12.0 ± 15.3 months, MAPK-wild type: median OS= 11.8 ± 7.4 months, p= 0.2*; FGFR1*-mut: median OS= 17.9 ± 28.3 months, *NF1*-mut: median OS= 33.8 ± 25.0 months, *KRAS*-mut: median OS= 9.0 ± 13.9 months, p= 0.3; female: median OS= 13.0 ± 17.5 months, male: median OS= 14.8 ± 12.1 months, p= 0.2). (**d**) The survival of patients with DMG-B of non-pontine localisations was as poor as the survival of patients with pontine DMG-B (DMG-B, non-pontine: median OS= 10.5 ± 8.1 months, DMG-B, pons: median OS= 11.0 ± 7.8 months, p= 0.7). (**e**) The survival of pediatric patients with *TP53*-mutant, non-pontine DMG-B was as poor as the survival of pediatric patients with *TP53*-mutant, pontine DMG-B (DMG-B, non-pontine, <18 years, *TP53*-mutant: median OS= 12.0 ± 8.9 months, DMG-B, pons, <18 years, *TP53*-mutant: median OS= 8.3 ± 4.5 months, p= 0.2). (**f**) In a univariate cox regression, age (paediatric vs. adult), tumour localisation (non-pontine vs. pontine), *PDGFRA*-status (balanced vs. amplified) and *TP53*-status (mutant vs. wt) significantly influence survival. However, these factors were dependent on the subtype attribution that has the largest effect on survival

"Integrated analyses reveal two molecularly and clinically distinct subtypes of H3 K27M-mutant diffuse midline gliomas with prognostic significance"

Acta Neuropathologica, Stegat L et al.
Corresponding author: Dr. Annika K. Wefers, Institute of Neuropathology, University Medical Center Hamburg-Eppendorf, Hamburg, Germany; present address: Department of Neuropathology, Regensburg University Hospital, Regensburg, Germany; annika.wefers@ukr.de
